# Supplementary material for: Trend analysis and projection of the gastric cancer disease burden in Taiwan during 1990–2021: An analysis of the global burden of disease study 2021
Source: PLoS One. 2025 Sep 12;20(9):e0331506. doi: 10.1371/journal.pone.0331506 (PMC12431411; doi:10.1371/journal.pone.0331506)
Supplement: S1 Table — (DOCX) [file pone.0331506.s001.docx]

**Supplementary Table**

**S1 Table. Joinpoint analysis of the changing trends of ASIR and ASPR of gastric cancer in Taiwan from 1990 to 2021**

|  | Age-standardized incidence rate | | | Age-standardized prevalence rate | | |
| --- | --- | --- | --- | --- | --- | --- |
| Gender | Period | APC(95%CI) | AAPC(95%CI) | Period | APC(95%CI) | AAPC(95%CI) |
| Both | 1990-1994 | 0.23(-1.93~2.44） | -1.99(-2.68~-1.3)* | 1990-1997 | 3.81(3~4.63)* | -1.2(-1.5~-0.89)* |
|  | 1994-1997 | 3.58(-3.23~10.88） |  | 1997-2005 | -1.67(-2.35~-0.98)* |  |
|  | 1997-2014 | -3.57(-3.78~-3.36）* |  | 2005-2014 | -4.22(-4.72~-3.72)* |  |
|  | 2014-2021 | -1.69(-2.42~-0.95）* |  | 2014-2021 | -1.6(-2.25~-0.94)* |  |
| Female | 1990-1994 | 0.71(-0.53~1.97） | -1.96(-2.57~-1.35)* | 1990-1997 | 4.33(3.76~4.91)* | -1.28(-1.55~-1)* |
|  | 1994-1997 | 4.91(0.52~9.49）* |  | 1997-2005 | -0.81(-1.39~-0.23)* |  |
|  | 1997-2000 | -4.22(-8.22~-0.06）* |  | 2005-2013 | -4.46(-5.06~-3.84)* |  |
|  | 2000-2005 | -2.04(-3.37~-0.69）* |  | 2013-2021 | -3.26(-3.84~-2.67)* |  |
|  | 2005-2013 | -4.02(-4.59~-3.45）* |  |  |  |  |
|  | 2013-2021 | -2.78(-3.37~-2.19）* |  |  |  |  |
| Male | 1990-1997 | 1.54(0.58~2.52）* | -1.71(-1.99~-1.43)* | 1990-1997 | 3.88(2.93~4.84)* | -0.94(-1.31~-0.58)* |
|  | 1997-2014 | -3.36(-3.58~-3.14）* |  | 1997-2006 | -1.95(-2.63~-1.27)* |  |
|  | 2014-2021 | -0.86(-1.58~-0.13）* |  | 2006-2014 | -4.04(-4.76~-3.31)* |  |
|  |  |  |  | 2014-2021 | -0.76(-1.52~0) |  |

ASIR, Age-standardized incidence rate; ASPR, Age-standardized prevalence rate; AAPC, Average annual percentage change; APC, Annual Percentage Change; CI, Confidence Interval. *P < 0.05.
