# Supplementary material for: Trend analysis and projection of the gastric cancer disease burden in Taiwan during 1990–2021: An analysis of the global burden of disease study 2021
Source: PLoS One. 2025 Sep 12;20(9):e0331506. doi: 10.1371/journal.pone.0331506 (PMC12431411; doi:10.1371/journal.pone.0331506)
Supplement: S2 Table — (DOCX) [file pone.0331506.s002.docx]

**Supplementary Table**

**S2 Table. Joinpoint analysis of the changing trends of ASMR and ASDR of gastric cancer in Taiwan from 1990 to 2021**

|  | Age-standardized mortality rate | | | Age-standardized DALYs rate | | |
| --- | --- | --- | --- | --- | --- | --- |
| Gender | Period | APC(95%CI) | AAPC(95%CI) | Period | APC(95%CI) | AAPC(95%CI) |
| Both | 1990-1994 | -1.22(-2.69~0.27) | -2.58(-3.26~-1.89)* | 1990-1994 | -1.8(-3.36~-0.22)* | -2.88(-3.59~-2.18)* |
|  | 1994-1997 | 3.33(-1.37~8.26) |  | 1994-1997 | 1.89(-3.02~7.06) |  |
|  | 1997-2000 | -6.25(-10.26~-2.06)* |  | 1997-2000 | -6.87(-11.07~-2.48)* |  |
|  | 2000-2015 | -3.65(-3.82~-3.48)* |  | 2000-2015 | -3.77(-3.94~-3.59)* |  |
|  | 2015-2018 | 0.12(-3.33~3.69) |  | 2015-2018 | 0.08(-3.28~3.56) |  |
|  | 2018-2021 | -3.66(-5.66~-1.63)* |  | 2018-2021 | -3.4(-5.34~-1.42)* |  |
| Female | 1990-1994 | -0.58(-1.86~0.71) | -2.4(-2.84~-1.95)* | 1990-1997 | -0.35(-0.95~0.26) | -2.78(-3.01~-2.56)* |
|  | 1994-1997 | 3.04(-1.23~7.49) |  | 1997-2013 | -3.8(-3.99~-3.61)* |  |
|  | 1997-2013 | -3.76(-3.95~-3.57)* |  | 2013-2021 | -2.84(-3.48~-2.19)* |  |
|  | 2013-2021 | -2.54(-3.18~-1.89)* |  |  |  |  |
| Male | 1990-1994 | -1.5(-3.35~0.38) | -2.4(-3.24~-1.55)* | 1990-1994 | -1.92(-3.67~-0.14)* | -2.67(-3.47~-1.87)* |
|  | 1994-1997 | 3.07(-2.85~9.35) |  | 1994-1997 | 2.29(-3.28~8.18) |  |
|  | 1997-2000 | -6.85(-11.72~-1.72)* |  | 1997-2000 | -7.5(-12.08~-2.69)* |  |
|  | 2000-2015 | -3.39(-3.6~-3.18)* |  | 2000-2015 | -3.57(-3.77~-3.36)* |  |
|  | 2015-2018 | 1.12(-3.06~5.47) |  | 2015-2018 | 1.19(-2.86~5.41) |  |
|  | 2018-2021 | -2.79(-5.03~-0.51)* |  | 2018-2021 | -2.83(-4.97~-0.64)* |  |

ASMR, Age-standardized mortality rate; ASDR, Age-standardized DALYs rate; AAPC, Average annual percentage change; APC, Annual Percentage Change; CI, Confidence Interval. *P < 0.05.
