# Supplementary material for: Trend analysis and projection of the gastric cancer disease burden in Taiwan during 1990–2021: An analysis of the global burden of disease study 2021
Source: PLoS One. 2025 Sep 12;20(9):e0331506. doi: 10.1371/journal.pone.0331506 (PMC12431411; doi:10.1371/journal.pone.0331506)
Supplement: S3 Table — (DOCX) [file pone.0331506.s003.docx]

**Supplementary Table**

**S3 Table. Projections of gastric cancer Incident Cases, Deaths, ASIR, and ASMR in Taiwan till 2036**

| Year | ASIR (per100,000) | | | ASMR (per100,000) | | | Number of incidences | | | Number of deaths | | | |
| --- | --- | --- | --- | --- | --- | --- | --- | --- | --- | --- | --- | --- | --- |
|  | Male | Female | Both | Male | Female | Both | Male | Female | Both | Male | Female | Both |  |
| 2022 | 16.40 | 6.69 | 11.18 | 12.56 | 5.30 | 8.56 | 3240 | 1550 | 4790 | 2475 | 1267 | 3742 |  |
| 2023 | 16.11 | 6.46 | 10.93 | 12.32 | 5.11 | 8.34 | 3265 | 1544 | 4809 | 2493 | 1265 | 3758 |  |
| 2024 | 15.82 | 6.24 | 10.69 | 12.08 | 4.94 | 8.12 | 3297 | 1538 | 4835 | 2513 | 1265 | 3778 |  |
| 2025 | 15.54 | 6.03 | 10.46 | 11.85 | 4.77 | 7.92 | 3338 | 1531 | 4869 | 2539 | 1264 | 3803 |  |
| 2026 | 15.28 | 5.82 | 10.25 | 11.64 | 4.61 | 7.73 | 3391 | 1524 | 4915 | 2573 | 1262 | 3835 |  |
| 2027 | 15.03 | 5.63 | 10.04 | 11.44 | 4.45 | 7.54 | 3455 | 1516 | 4971 | 2613 | 1259 | 3872 |  |
| 2028 | 14.78 | 5.44 | 9.83 | 11.23 | 4.30 | 7.36 | 3531 | 1510 | 5041 | 2660 | 1256 | 3916 |  |
| 2029 | 14.52 | 5.26 | 9.63 | 11.03 | 4.15 | 7.18 | 3624 | 1505 | 5129 | 2718 | 1256 | 3974 |  |
| 2030 | 14.28 | 5.08 | 9.43 | 10.83 | 4.01 | 7.01 | 3738 | 1501 | 5239 | 2786 | 1258 | 4044 |  |
| 2031 | 14.05 | 4.92 | 9.25 | 10.65 | 3.88 | 6.85 | 3875 | 1498 | 5373 | 2867 | 1260 | 4127 |  |
| 2032 | 13.83 | 4.76 | 9.07 | 10.47 | 3.75 | 6.69 | 4038 | 1496 | 5534 | 2957 | 1264 | 4221 |  |
| 2033 | 13.60 | 4.60 | 8.90 | 10.29 | 3.63 | 6.54 | 4231 | 1496 | 5727 | 3060 | 1269 | 4329 |  |
| 2034 | 13.37 | 4.45 | 8.72 | 10.11 | 3.50 | 6.38 | 4467 | 1498 | 5965 | 3180 | 1276 | 4456 |  |
| 2035 | 13.15 | 4.31 | 8.56 | 9.94 | 3.39 | 6.24 | 4752 | 1501 | 6253 | 3319 | 1285 | 4604 |  |
| 2036 | 12.94 | 4.17 | 8.39 | 9.77 | 3.28 | 6.09 | 5098 | 1505 | 6603 | 3478 | 1294 | 4772 |  |

ASIR, Age-standardized incidence rate; ASMR, Age-standardized mortality rate
