# Supplementary material for: Trend analysis and projection of the gastric cancer disease burden in Taiwan during 1990–2021: An analysis of the global burden of disease study 2021
Source: PLoS One. 2025 Sep 12;20(9):e0331506. doi: 10.1371/journal.pone.0331506 (PMC12431411; doi:10.1371/journal.pone.0331506)
Supplement: Attachment — Submitted filename: Response to Reviewers.docx [file pone.0331506.s005.docx]

**List of responses**

Dear Editors and Reviewers:

Thank you for your letter and for the reviewers’ comments concerning our manuscript entitled “Trend analysis and projection of the gastric cancer disease burden in Taiwan during 1990 - 2021: An analysis of the global burden of disease study 2021” (Manuscript Number: PONE-D-25-03556). Those comments are all valuable and very helpful for revising and improving our paper, as well as the important guiding significance for our research. We have revised the manuscript, according to the comments and suggestions of reviewers and the editor, and responded, point by point, to the comments as listed below, which we hope will meet with approval. Revised portions are marked in red in the paper. The main corrections in the paper and the responses to the reviewer’s comments are as follows:

**Response to Journal Requirements**

**1. Please ensure that your manuscript meets PLOS ONE's style requirements, including those for file naming. The PLOS ONE style templates can be found at**

**https://journals.plos.org/plosone/s/file?id=wjVg/PLOSOne_formatting_sample_main_body.pdf and**

[**https://journals.plos.org/plosone/s/file?id=ba62/PLOSOne_formatting_sample_title_authors_affiliations.pdf**](https://journals.plos.org/plosone/s/file?id=ba62/PLOSOne_formatting_sample_title_authors_affiliations.pdf)

The authors’ answer: We have carefully reviewed PLOS ONE**'s** style requirements and ensured that our revised manuscript adheres to all specified guidelines, including file naming conventions and formatting standards.

**2. Thank you for stating the following financial disclosure: This work was supported by grants to the Young and Middle-aged Talents Training Project of Fujian Provincial Health Technology Project (2021GGA021); Startup Fund for Scientific Research, Fujian Medical University (2020QH1098); Joint Funds for the National Key Clinical Specialty Construction Projects of Fujian Province, China (Grant No. 2023 − 1594) and Minimally Invasive Medical Center of Fujian, China (Grant No. [2017]171).Please state what role the funders took in the study. If the funders had no role, please state: "The funders had no role in study design, data collection and analysis, decision to publish, or preparation of the manuscript." If this statement is not correct you must amend it as needed. Please include this amended Role of Funder statement in your cover letter; we will change the online submission form on your behalf.**

The authors’ answer: Thank you for your guidance on the funder role statement. We confirm the following regarding the involvement of the funding agencies in this study: the Young and Middle-aged Talents Training Project of Fujian Provincial Health Technology Project (2021GGA021), which played a role in the decision to publish. Additionally, the Startup Fund for Scientific Research Fujian Medical University (2020QH1098), Joint Funds for the National Key Clinical Specialty Construction Projects of Fujian Province, China (Grant No. 2023 − 1594) and Minimally Invasive Medical Center of Fujian, China (Grant No. [2017]171) provided support for this work, contributing to data collection and analysis.

**3. Please include your full ethics statement in the ‘Methods’ section of your manuscript file. In your statement, please include the full name of the IRB or ethics committee who approved or waived your study, as well as whether or not you obtained informed written or verbal consent. If consent was waived for your study, please include this information in your statement as well.**

The authors’ answer: We have carefully revised the manuscript to include the full ethics statement in the *Methods* section, as specified. Details are as follows: All data in this study were sourced from the publicly accessible GBD database and subjected to secondary analysis, rendering ethical approval and informed consent unnecessary (lines 149-152).

**4. Thank you for uploading your study's underlying data set. Unfortunately, the repository you have noted in your Data Availability statement does not qualify as an acceptable data repository according to PLOS's standards. At this time, please upload the minimal data set necessary to replicate your study's findings to a stable, public repository (such as figshare or Dryad) and provide us with the relevant URLs, DOIs, or accession numbers that may be used to access these data. For a list of recommended repositories and additional information on PLOS standards for data deposition, please see** [**https://journals.plos.org/plosone/s/recommended-repositories**](https://journals.plos.org/plosone/s/recommended-repositories)**.**

The authors’ answer: Thank you for your feedback regarding the data availability. We appreciate the opportunity to clarify the data deposition for our study. The underlying data used in this study were obtained from the publicly accessible Global Burden of Disease 2021 (GBD 2021) database, which is a stable, public repository meeting PLOS's standards for data deposition. The data can be accessed via the following URL: <http://ghdx.healthdata.org/gbd-results-tool>.We have updated the Data Availability statement in the revised manuscript to explicitly reference the GBD 2021 database and its URL. The specific steps for downloading the data are as follows: for the cause, “Stomach cancer”; for measurements, “incidence, prevalence, deaths and DALYs”; for location, “China and Taiwan (Province of China)”; for years, “1990–2021”; for metrics, “number, percent, and rate”; for sex, “both, male and female”. Age stratification included: <5 years,5-9 years,10-14 years,15-19 years,20-24 years,25-29 years,30-34 years,35-39 years,40-44 years,45-49 years,50-54 years,55-59 years,60-64 years,65-69 years,70-74 years,75-79 years,80-84 years,85-89 years,90-94 years,95+years, All ages, Age-standardized.

**5. When completing the data availability statement of the submission form, you indicated that you will make your data available on acceptance. We strongly recommend all authors decide on a data sharing plan before acceptance, as the process can be lengthy and hold up publication timelines. Please note that, though access restrictions are acceptable now, your entire data will need to be made freely accessible if your manuscript is accepted for publication. This policy applies to all data except where public deposition would breach compliance with the protocol approved by your research ethics board. If you are unable to adhere to our open data policy, please kindly revise your statement to explain your reasoning and we will seek the editor's input on an exemption. Please be assured that, once you have provided your new statement, the assessment of your exemption will not hold up the peer review process.**

The authors’ answer: Thank you for your guidance on the data availability policy. We appreciate the opportunity to clarify our data sharing plan and address the requirements promptly. The study utilizes publicly available data from the Global Burden of Disease 2021 (GBD 2021) database, which is accessible via <http://ghdx.healthdata.org/gbd-results-tool>. As this data is already freely available, no access restrictions apply, and it fully complies with PLOS's open data policy. We have amended the data availability statement to clarify that the data sharing protocol will be determined before manuscript acceptance.

**6. Please include a new copy of Tables 1 and 2 in your manuscript; the current table is difficult to read. Please follow the link for more information:**

[**https://blogs.plos.org/plos/2019/06/looking-good-tips-for-creating-your-plos-figures-graphics/**](https://blogs.plos.org/plos/2019/06/looking-good-tips-for-creating-your-plos-figures-graphics/)

The authors’ answer: Thank you for the feedback on the readability of Tables 1 and 2. We have revised the table formats to enhance clarity, by the PLOS guidelines for figure and table presentation. The revised Table 1 has been inserted into the corresponding section of the manuscript (line 570), and Table 2 has been moved to the supplementary materials as Supplementary Tables 1 and 2.

**7. We notice that your supplementary table is included in the manuscript file. Please remove them and upload them with the file type 'Supporting Information'. Please ensure that each Supporting Information file has a legend listed in the manuscript after the references list.**
The authors’ answer: Thank you for your meticulous guidance on the supplementary materials. In the revised manuscript, we have revised the supplementary tables as required to ensure full compliance with the submission guidelines of *PLOS ONE*.

**Response to Reviewer 1**

**Reviewer #1 Comment 1: It would be helpful to clarify whether the BAPC model takes into account dynamic factors that may influence the future burden of gastric cancer, such as changes in dietary habits. Please specify the assumptions underlying the BAPC model and discuss potential sources of bias in extrapolating the results.**

The authors’ answer: Thank you for your valuable suggestion. The Bayesian Age-Period-Cohort (BAPC) model correlates in predicting future disease burden trends by analyzing age, period, and cohort effects in historical data. For model construction, we primarily analyzed existing epidemiological data and historical trends from the Global Burden of Disease (GBD) database. While the database includes partial dietary risk factors for gastric cancer (e.g., high-sodium diets), it lacks risk data on other dietary habits. We also recognize that changes in dynamic factors such as dietary habits are complex and may be influenced by the combined effects of socioeconomic, cultural, and health awareness factors, which affect model predictions to some extent. The BAPC model is based on the following assumptions: 1) Independence assumption: Effects between different ages, periods, and cohorts are assumed to be mutually independent within the model. 2) Smoothness assumption: Changes in age, period, and cohort effects are assumed to be smooth rather than abrupt. 3) Data representativeness assumption: The data used are considered representative of the overall study population. We summarized these assumptions in the methods section and presented the underlying equations (lines 136–145). Potential sources of bias may arise when extrapolating BAPC model results to the future: For example, the limited period of data may lead to inaccurate capture of long-term trends by the model, and changes in external factors such as dietary habits have not yet fully manifested their impact on gastric cancer incidence. Additionally, model assumptions are limited: interactions between different age groups (e.g., transmission of dietary habits within families) may introduce biases into predicted results. Uncertainties in external dynamic factors—including changes in dietary habits, socioeconomic development, and advances in medical technology—also contribute to biases in future disease burden projections. We have addressed in the limitations section (lines 412–420).

**Reviewer #1 Comment 2: While the manuscript references an APC model, it does not detail how the inherent collinearity among age, period, and cohort variables was handled. Providing additional methodological details on how you addressed linear dependence will bolster the credibility of your findings.**

The authors’ answer: We appreciate the reviewer’s valuable suggestion. The inherent collinearity between age, period, and cohort (APC) variables is of substantial importance and represents a key component that demands detailed clarification in our research. These three variables exhibit a linear dependence—specifically, "period = age + cohort"—which introduces instability in model parameter estimation and complicates interpretation. In the present study, we utilized the intrinsic estimator to address collinearity by applying linear constraints to the parameter space, yielding unique, stable estimates with minimal bias. Given the specific characteristics of the intrinsic estimator, we stratified ages into 17 groups (15–19 years to ≥95 years) at 5-year intervals, alongside 22 birth cohorts (1897–1901 to 2002–2006) and 6 periods (1992–1996 to 2017–2021), to present gastric cancer incidence and mortality for specific ages, periods, and birth cohorts. Details are provided in the "Age–Period–Cohort Analysis" section of the Methods.

**Reviewer #1 Comment 3: The study predicts a decrease in both the ASIR and ASMR for gastric cancer in Taiwan by 2036. However, despite the overall decline in these rates, the number of incident cases is projected to rise, particularly due to Taiwan's aging population. Given this trend, I recommend expanding the discussion to include tailored interventions for this demographic. This would make the findings more actionable and relevant for public health policymakers.**

 The authors’ answer: Thank you for this insightful suggestion. In the revised "Discussion" section, we added content outlining targeted interventions, including precise management of risk factors; enhanced gastroscopic screening, early intervention, and multidisciplinary collaboration for high-risk populations (elderly individuals); development of personalized treatment plans; and increased resource investment in primary care, alongside upgrades to gastroscopic equipment and technical capabilities.

**Reviewer #1 Comment 4: On Line 167, the term “AMPR” appears, which should presumably be “ASMR”.**

The authors’ answer: We appreciate you identifying this error. You are correct that "AMPR" here is a typo and should be "ASMR." This will be corrected in the revised manuscript (line 163), and we will carefully review the entire text to ensure no similar errors are overlooked.

**Response to Reviewer 2**

**Reviewer #2 Comment: The conclusion of The Trend analysis and projection of the gastric cancer disease burden in Taiwan during 1990 - 2021: An analysis of the global burden of disease study is stable. The figures are needed to revised. There are some grammatical errors to corrected.**

The authors’ answer: We appreciate your review of our study and your valuable comments. All figures and tables will undergo careful inspection and revision to ensure they clearly and accurately reflect the study findings. Specifically, minor issues in the legend of Figure 6 have been addressed, with the revised figure reuploaded. Regarding the incomplete presentation of Tables 1 and 2, revisions have been made: the revised Table 1 has been inserted into the manuscript at the corresponding position (line 570), and Table 2 has been moved to the supplementary materials as Supplementary Tables 1 and 2. Additionally, a colleague whose native language is English has proofread and revised the entire manuscript to ensure fluent, accurate language that conforms to English expression conventions.

**Response to Reviewer 3**

**Reviewer #3 Comment 1: Lines 68 – 72. Please add the following two references to strengthen the statement. Lee YC et al. Screening for Helicobacter pylori to Prevent Gastric Cancer: A Pragmatic Randomized Clinical Trial. JAMA 2024 Nov 19;332(19):1642-1651. Liou JM et al. Screening and eradication of Helicobacter pylori for gastric cancer prevention: the Taipei global consensus. Gut 2020 Dec;69(12):2093-2112.**

The authors’ answer: We appreciate your valuable suggestions. The two additional references you recommended will significantly strengthen our argument that Helicobacter pylori infection is a risk factor for gastric cancer. These references will be cited in the manuscript (lines 59–63). Thank you again for your input, which will enhance the comprehensiveness and persuasiveness of our study.

**Reviewer #3 Comment 2: According to the study authored by Liu PC et al. Disease Burden of 30 Cancer Groups in Taiwan from 2000 to 2021. J Epidemiol Glob Health 2025 Apr 22;15(1):62. Regarding stomach cancer, the age-standardized rate for mortality in 2000 was estimated to be 13.40 per 100,000 population for both sexes. Twenty-one years later in 2021, it was 6.22/100,000. It would be much appreciated if the authors can cite and discuss the subtle differences between the current study and that from the Liu’s study.**

The authors’ answer: Thank you for this insightful suggestion. We will cite the study by Liu PC et al. and compare the differences between the two studies in the Discussion section. Specifically, there are minor discrepancies between our study and that of Liu PC et al. regarding the age-standardized mortality rate (ASMR) and age-standardized disability rate (ASDR) of gastric cancer in Taiwan in 2021. This variation stems from differing data sources: the Taiwan Cancer Registry is based on hospital registry data in Taiwan, while cancer burden estimates in the GBD study derive from cancer registries, vital statistics, and autopsy data, among other sources (lines 306-311).

**Reviewer #3 Comment 3: Table 1: Please remove ‘(Province of China)’ following Taiwan to avoid introducing political or ideological implications into the study. We also recommend removing similar references throughout the manuscript to maintain neutrality and ensure appropriateness for an international readership. The term ‘mainland China’ may be retained to reflect goodwill and provide clarity where relevant. We believe that such adjustments may encourage broader acceptance and citation of your work among Taiwanese scholars. A win-win strategy.**

The authors’ answer: We appreciate your attention to our study and your valuable comments. We fully understand and respect your suggestion to avoid introducing political or ideological implications, ensuring our research is presented to international readers in a neutral and objective manner. In Table 1 and throughout the manuscript, we will remove "(Province of China)" from "Taiwan (Province of China)," retaining only "Taiwan," and will carefully review the entire text to ensure no similar expressions remain. We agree with your observation that retaining "mainland China" in certain contexts can reflect goodwill and provide clarity. When specific reference to the mainland region of China is necessary in the text, we will use "mainland China" to ensure the accuracy and completeness of the research. These adjustments will ensure our study avoids unnecessary political connotations.

**Reviewer #3 Comment 4: Line 520: please modify the word, ‘province’ to territory, or simply island.**
The authors’ answer: Thank you very much for pointing this out. We will follow your suggestion to revise "province" to "territory" to ensure the accuracy and neutrality of the expression (line 435).

**Reviewer #3 Comment 5: Do the authors have the capacity to perform a health disparities analysis to examine why one population (mainland China) bears a higher disease burden than another (Taiwan), by disentangling the effects of demographic composition, disease risk, and other contributing factors?**

The authors’ answer: We thanks for your insightful suggestion. In response, we reviewed the age-standardized rates of DALYs, deaths, incidence, and prevalence between mainland China and Taiwan from 1990 to 2021, stratified by sex (Figure 1). As shown in the figure, mainland China consistently exhibits a higher disease burden across all indicators and in both sexes compared to Taiwan. This disparity is particularly pronounced for DALYs and mortality, indicating not only a higher occurrence of disease but also worse outcomes and/or more limited access to effective care. We believe this observed disparity is likely attributable to several interacting factors: (1) Demographic composition: Although the rates are age-standardized to minimize the impact of differing age structures, mainland China’s much larger and more heterogeneous population may exacerbate disparities arising from uneven distribution of healthcare resources. (2) Disease risk factors: The higher burden in mainland China may reflect greater exposure to known risk factors (e.g., smoking, occupational and environmental hazards, and dietary patterns) that remain more prevalent in mainland China than in Taiwan. (3) Healthcare access and quality: Taiwan’s implementation of universal health coverage and its relatively high standard of medical care likely contribute to earlier detection, better treatment, and improved survival outcomes. In contrast, mainland China continues to face challenges with healthcare accessibility and quality, particularly in rural and underdeveloped regions. We acknowledge that a formal health disparities analysis to disentangle and quantify the relative contributions of these factors (e.g., via decomposition techniques such as the Kitagawa–Blinder–Oaxaca method) would provide further valuable insights. However, this level of analysis falls beyond the scope of the present study. We appreciate the reviewer highlighting this important perspective and will consider pursuing it in future research endeavors.

**Figure 1. Trends in age-standardized rates of DALYs, deaths, incidence, and prevalence for mainland China and Taiwan, 1990–2021, stratified by sex. (**Each panel shows the age-standardized rate (per 100,000 population) for one indicator (rows: DALYs, deaths, incidence, prevalence) and one sex category (columns: both sexes combined, female, male). Red points and lines represent mainland China, and blue points and lines represent Taiwan. Over the study period, mainland China consistently exhibited higher rates across all indicators and sexes compared to Taiwan, with particularly marked differences in DALYs and mortality).

**Reviewer #3 Comment 6: Line 388, a reference is needed following the statement.**

The authors’ answer: We appreciate your attention to our study and your valuable comments. We will add appropriate references following this statement to support the argument (lines 317).

We tried our best to improve the manuscript and made some changes to the manuscript. These changes will not influence the content and framework of the paper. Here we did not list the changes, but marked them in red in the revised paper, and we also uploaded a clean version in the attachment.

We appreciate the warm work of the editors and reviewers warm work earnestly, and hope that the correction will meet with approval.

Once again, thank you very much for your comments and suggestions.

Thank you and best regards.

Yours sincerely,

Xiaohuang Yang

1. Department of Gastroenterology and Fujian Institute of Digestive Disease, Fujian Medical University Union Hospital, Fuzhou, Fujian, China

2. Department of Gastroenterology, Zhangzhou Affiliated Hospital of Fujian Medical University, Zhangzhou, Fujian, China

Email: [dryangxh@163.com](mailto:dryangxh@163.com);
